# Mixed messages? Exposure to reports about alcohol’s suggested cardiovascular effects and hazardous alcohol use: a cross-sectional study of patients in cardiology care

**DOI:** 10.1186/s12889-024-18783-5

**Published:** 2024-05-13

**Authors:** Paul Welfordsson, Anna-Karin Danielsson, Caroline Björck, Bartosz Grzymala-Lubanski, Matthias Lidin, Ida Haugen Löfman, Sara Wallhed Finn

**Affiliations:** 1 Department of Global Public Health, Karolinska Institutet, Solna, 113 65 Sweden; 2 Department of Women’s and Children’s Health, Akademiska Sjukhuset, Uppsala University, Uppsala, Sweden; 3 Centre for Research and Development, Region Gävleborg, Gävle, Sweden; 4 Department of Public Health and Clinical Medicine, Umeå University, Umeå, Sweden; 5 Department of Medicine, Unit of Cardiology, Karolinska Institutet, Solna, Stockholm, Sweden; 6 Department of Cardiology, Heart and Vascular Center, Karolinska University Hospital, Stockholm, Sweden; 7 Department of Medicine, Unit of Cardiology, Karolinska Institutet, Huddinge, Stockholm, Sweden; 8 Unit of Clinical Alcohol Research, Institute of Clinical Research, University of Southern Denmark, Odense, Denmark; 9 Centre for Dependency Disorders, Stockholm, Sweden

**Keywords:** Cardiology, Information sources, Positive effects, Association, Hazardous alcohol use, Public health

## Abstract

**Background:**

Hazardous alcohol use is a leading risk factor for disability and death, yet observational studies have also reported reduced cardiovascular disease mortality among regular, low-level drinkers. Such findings are refuted by more recent research, yet have received significant media coverage. We aimed to explore: (1) how patients with cardiovascular diseases access health information about moderate drinking and cardiovascular health; (2) the perceived messages these sources convey, and (3) associations with own level of alcohol use.

**Methods:**

We conducted a cross-sectional survey of patients in cardiology services at three hospitals in Sweden. The study outcome was hazardous alcohol use, assessed using the AUDIT-C questionnaire and defined as ≥ 3 in women and ≥ 4 in men. The exposure was accessing information sources suggesting that moderate alcohol consumption can be good for the heart, as opposed to accessing information that alcohol is bad for the heart. Health information sources were described using descriptive statistics. Gender, age and education were adjusted for in multiple logistic regression analyses.

**Results:**

A total of 330 (66.3%) of 498 patients (mean age 70.5 years, 65% males) who had heard that drinking moderately can affect the heart described being exposed to reports that moderate alcohol use can be good for the heart, and 108 (21.7%) met criteria for hazardous alcohol use. Health information sources included newspapers (32.9%), television (29.2%), healthcare staff (13.4%), friends/family (11.8%), social media (8.9%) and websites (3.7%). Participants indicated that most reports (77.9%) conveyed mixed messages about the cardiovascular effects of moderate drinking. Exposure to reports of healthy heart effects, or mixed messages about the cardiovascular effects of alcohol, was associated with increased odds of hazardous alcohol use (OR = 1.67, 95%CI = 1.02–2.74).

**Conclusions:**

This study suggests that many patients in cardiology care access health information about alcohol from media sources, which convey mixed messages about the cardiovascular effects of alcohol. Exposure to reports that moderate drinking has protective cardiovascular effects, or mixed messages about the cardiovascular effects of alcohol, was associated with increased odds of hazardous alcohol use. Findings highlight a need for clear and consistent messages about the health effects of alcohol.

**Supplementary Information:**

The online version contains supplementary material available at 10.1186/s12889-024-18783-5.

## Introduction

Hazardous alcohol use increases the risk of ischaemic heart disease, arrhythmia, heart failure and stroke [1, 2], and is a leading risk factor for disability and death [1]. However, observational studies have also reported reduced cardiovascular disease mortality among regular, low-level drinkers [3, 4]. These findings are controversial, and remain an active area of debate [5], being refuted by more recent research that utilize genetic predisposition for alcohol use rather than self-report, so called Mendelian randomization studies [6]. A recent meta-analysis of 107 observational studies also reported that moderate daily alcohol intake is not significantly associated with a reduction in all-cause mortality [7]. Similarly, the Global Burden of Disease study indicates that moderate alcohol consumption increases overall risks to health, including cancer risks [1]. These risks appear to be greatest among young adults [8]. Despite a shift towards alcohol use being considered an important modifiable cardiovascular risk factor [9], the purported ‘healthy heart’ effects of moderate drinking have received extensive media coverage and appear to have reached the attention of patients with cardiovascular diseases (CVDs) [10].

Hazardous alcohol use – a pattern of drinking that increases a person’s risk of harm – may involve excessive regular consumption, binge drinking, or both. The burden of hazardous alcohol use and related harms are generally greater in men than women [1, 11], including among patients in hospital settings [12, 13]. Age is also an important factor when considering alcohol use, with a lower prevalence of hazardous drinking reported among patients in cardiology care aged 70–79, compared with patients aged 50–59 [12], an observation that may be partly related to increased morbidity among older adults. Hazardous drinking has generally been reported to be associated with lower education [14], although a study involving cardiology inpatients did not report significant differences according to educational level [12].

The health belief model (HBM) is a widely-used theoretical framework that aims to explain and predict health behaviours according to beliefs and expectations [15]. Since its conception more than 50 years ago, a considerable body of evidence has accumulated for the HBM, indicating its ongoing usefulness as a basis for understanding and influencing behaviours [16]. The HBM proposes that individuals who believe that a particular lifestyle habit is associated with health benefits are more likely to engage in that habit. In contrast, those who believe that the same habit is risky are suggested to be less inclined to engage in it. In addition to formal learning, such health beliefs may be acquired and shaped by subjective experiences, social contexts and media sources – a phenomenon known as lay epidemiology [17, 18]. Lay epidemiology suggests that individuals reach an overall interpretation of risk based on a balance between potential benefits, such as perceived social advantages or possible beneficial health effects, and perceived negative effects. According to lay epidemiology theory and the HBM, exposure to information sources that propose a healthy heart effect may shift the perceived risk–benefit balance of moderate drinking in favour of heavy alcohol consumption and increase an individuals’ propensity to drink regularly.

In spite of widespread reports of alcohol’s purported cardiovascular effects, there has been remarkably little research on how risks of alcohol consumption are perceived by adults with CVDs [10, 19]. Studies to date are restricted to the US, limiting generalizability to other contexts. One survey, conducted during 2013–2014, found that 31% of participants were unsure whether alcohol affects the heart, while 30% viewed alcohol as good for the heart and 39% as unhealthy for the heart [19]. A more recent study of 290 patients hospitalized with acute cardiac events found that 69% of respondents had heard that that moderate alcohol consumption is good for the heart [10]. Most respondents who reported having heard about beneficial cardiovascular effects of alcohol indicated that they had done so via lay press [10, 19], followed by family/friends. Twelve patients (4%) reported increasing their alcohol consumption in light of these suggested positive cardiovascular effects [10].

There is a need to further elucidate the association between exposure to information sources suggesting cardioprotective effects of moderate drinking, and alcohol use among individuals with cardiovascular diseases (CVDs). We therefore conducted a cross-sectional survey of patients in cardiology services, aiming to explore how patients with CVDs access health information about moderate drinking and cardiovascular health, the perceived messages that these sources convey, and possible associations with own alcohol use. More specifically, we aimed to answer the following research questions:

1. Which information sources do patients access information about moderate alcohol and cardiovascular health from?
2. What perceived messages do these sources convey about moderate alcohol consumption and cardiovascular health?
3. To what extent is exposure to information suggesting that alcohol consumption is good for the heart associated with own level of alcohol use?

## Methods

### Study design

This cross-sectional study adhered to the Reporting of Observational Studies in Epidemiology (STROBE) statement (Supplementary material 1) [20]. A study protocol and data analysis plan are publicly available [21]. Approval was granted by the Swedish Ethical Review Authority (2022-02059-01). Informed consent was obtained from all participants prior to recruitment.

### Setting

Participants were recruited from cardiology services at three hospitals in Sweden: Karolinska University hospital, Stockholm (specialized centre in a large city); Gävle hospital (general hospital in a medium-sized town) and Falun hospital (general hospital in a small-town/rural area) [22]. Data was collected between October 2022–August 2023.

### Participants

**Fig. 1:**
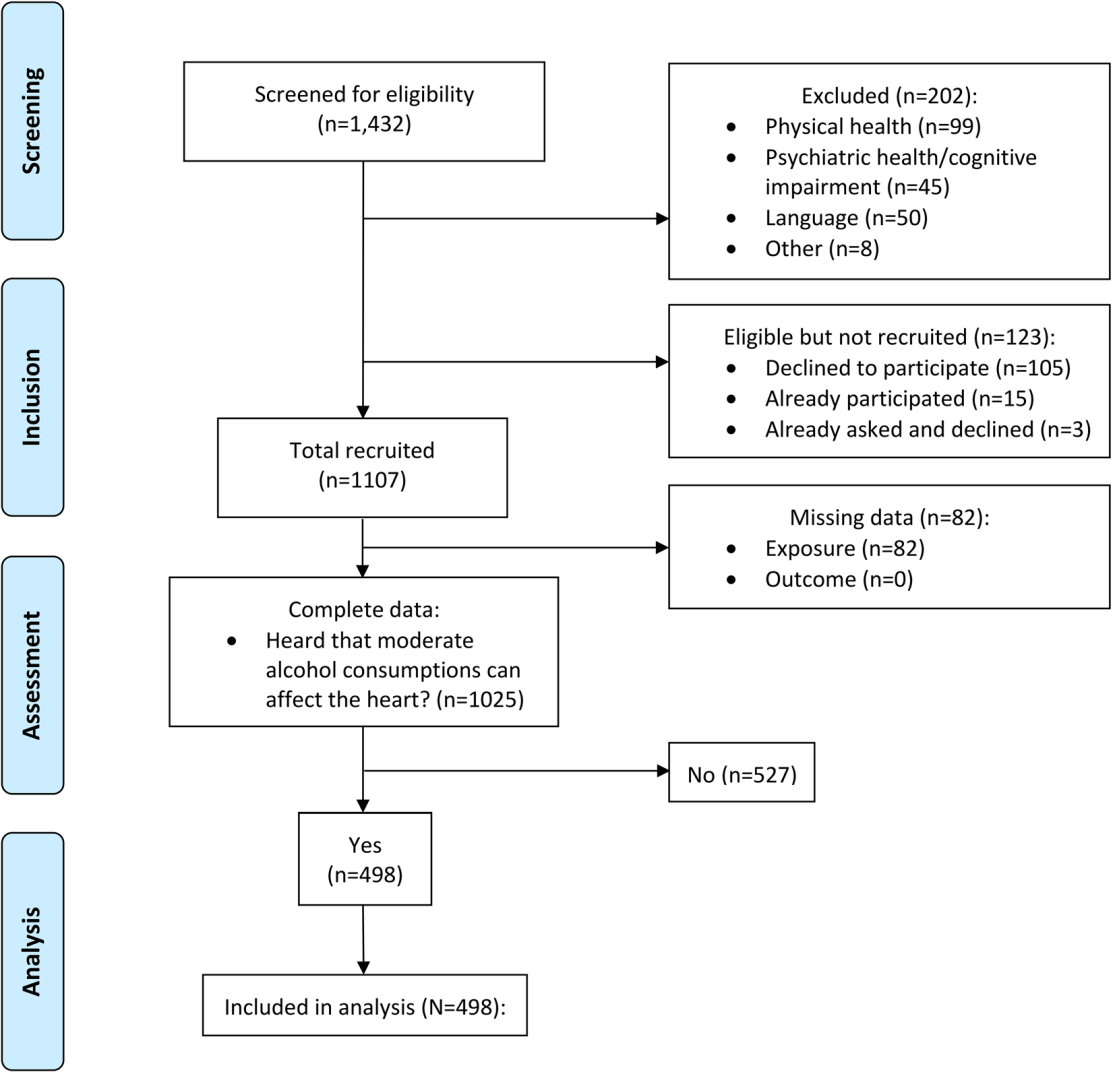
Flow of participants through the study

Consecutive patients were screened and recruited by trained assessors (PhD student, registered nurses) on cardiology wards at participating centres. We also recruited a convenience sample of patients (those who arrived **≥** 15 min early) from the waiting room at the outpatient cardiology clinic in Gävle and, where staffing levels permitted, at the ambulatory cardiology clinic in Stockholm. Eligibility criteria included: age **≥** 18 years; fluent in Swedish or English; no physical, cognitive or mental health problems preventing survey completion (e.g., dementia, delirium, agitation, or advanced end-of life-care), no infectious diseases necessitating barrier nursing. The flow of participants through the study is illustrated in Fig. 1.

### Study outcome

**Hazardous alcohol use** was assessed using the AUDIT-C questionnaire – a three-item World Health Organization alcohol use screening instrument – and defined as a binary (yes/no) variable using established cut-offs of ≥ 3 in women and ≥ 4 in men [23]. All participants were initially asked whether they had consumed alcohol (yes/no) during the previous year. Those who answered ‘yes’ were asked to complete AUDIT-C. Those who answered ‘no’ were coded as no hazardous alcohol use.

### Exposure

**Healthy heart effect** was a binary variable: Participants who indicated that they had heard that moderate alcohol consumption can be ‘good for the heart’ or ‘both good and bad for the heart’ were categorised as being exposed to reports of a healthy heart effect. Conversely, those who had heard reports that moderate alcohol consumption is ‘bad for the heart’ were categorised as not exposed to a healthy heart effect.

### Other variables

**Health information sources** were self-reported from multiple options including: ‘healthcare staff’; ‘newspaper’; ‘TV’; ‘friend/family’; ‘social media’; ‘website’; ‘don’t know’. Participants were asked to (1) indicate all sources from which they had heard that moderate alcohol consumption can affect the heart and (2) indicate whether each source reported that moderate alcohol consumption is ‘good for the heart’, ‘bad for the heart’, or ‘both that it can be bad and good for the heart`. **Gender** identity was self-reported as one of three categories: ‘male’, ‘female’, or ‘other’. **Age** was reported in years and categorized as age groups: 18**–**44; 45**–**69; ≥70 years. **Education** was self-reported as one of four categories: ‘not completed primary school’; ‘completed primary school’; ‘completed secondary school’, or ‘completed higher education’.

### Data sources/measurement

We developed an electronic survey using REDCap (Supplementary material 2) and piloted this with cardiology patients in July 2022. Trained assessors then approached patients at the three study sites. Those eligible and consenting either: (1) completed the survey under the supervision of assessors, using a study tablet or their mobile phone, (2) responded to questions in a face-to-face interview, or (3) completed a hybrid version of 1.) and 2.), according to individual participant preference.

### Bias

To minimize selection bias, consecutive patients were included wherever possible. Assessors emphasized that participation was anonymous, confidential, and would not affect medical treatment. No payment or other incentives for participation were offered. To maximize inclusivity, all study materials were available in both English and in Swedish.

### Study size

Overall study size was based on a sample size calculation that aimed to estimate the minimum number of participants required to detect differences in alcohol health literacy [21]. As described in our study protocol, that calculation used reported knowledge of UK national alcohol guidelines from the Alcohol Toolkit study [24], and suggested that 410 participants were required. The current report, however, relates to specific aspects of the larger survey, namely questions about health information sources. We did not perform *a priori* sample size calculations for the specific variables of interest in this report.

### Statistical methods

We calculated survey response rates, along with the overall proportion of participants who had heard that moderate alcohol consumption can affect the heart. We used chi-squared tests to examine for differences according to whether participants had heard that moderate alcohol consumption can affect the heart. To examine for possible ‘healthy heart effects’, analyses were subsequently limited to participants who had heard that moderate drinking can affect the heart. Descriptive statistics were calculated and assessed for differences, using chi-squared tests reported with Cramér’s V statistics and standardised residuals. We calculated frequencies and percentages for **health information sources** and messages about moderate drinking. We excluded ‘don’t know’ responses and ‘other’ information sources when reporting this data. We then assessed the association between exposure to information sources reporting a perceived **healthy heart effect** and **hazardous alcohol use**; first using univariate logistic regression, then via multiple logistic regression models, adjusting sequentially for gender, age group and education. Odds ratios (ORs) were reported with 95% confidence intervals (CIs). Participants with missing questionnaire data, or who responded ‘don’t know’ for the study exposure, were excluded. All analyses were conducted in StataSE v17.

### Sensitivity analyses

To explore the effect of separating participants who had not consumed alcohol during the previous year from those who consumed alcohol at non-hazardous levels, we conducted ordinal logistic regression analyses using a three-level outcome variable with the following categories: (1) Abstainers (No alcohol consumed during previous year), (2) No hazardous alcohol use (consumed alcohol during last years at non-hazardous levels, (3) Hazardous alcohol use (using identical AUDIT-C cut-off to those in the main analysis).

## Results

From a total of 1230 eligible patients, 1107/1230 (90.0%, 66% males) participated in the survey and 1025/1230 (83.0%) responded to questions about encountering reports that moderate alcohol consumption can affect the heart (Gävle outpatient clinic, *n* = 310; Gävle inpatients, *n* = 309; Falun inpatients, *n* = 202; Stockholm inpatients, *n* = 140; Stockholm ambulatory clinic, *n* = 64). Of these, 498/1025 (48.6%) patients had heard that drinking moderately can affect the heart (Supplementary material 3). Older participants (*p* = .042), and those who did not meet criteria for hazardous drinking (*p* = .005) were more likely to have heard that moderate drinking can affect the heart. Among those who had heard that drinking alcohol can affect the heart, 330/498 (66.3%) indicated that they had heard reports of a healthy heart effect. Participants exposed to information sources reporting a healthy heart effect tended to be more highly educated than those who reported hearing that drinking moderately is bad for the heart (*p* = .010); Among those exposed to reports of a healthy heart effect, 30.6% had university level education, compared to 22.0% among those who reported hearing that moderate drinking is bad for the heart. Overall, 108 (21.7%) of those included in the analyses met criteria for hazardous alcohol use: 24.2% of those exposed to reports of a healthy heart effect and 16.7% among those not exposed. Characteristics of study participants are further described in Table 1.

**Table 1:** Characteristics of study participants, by exposure to information sources reporting that moderate alcohol consumption has healthy heart effects (*N* = 498)

	Exposed to reports of a healthy heart effect, *n* = 330 (66.3%): *n* (%, standardised residual)	Accessed reports that moderate drinking is bad for the heart (not exposed), *n* = 168 (33.7%): *n* (%, standardised residual)	*p*-value	Cramér’s V
**Age group:**			0.804	0.030
18–44	17 (5.2, -0.21)	10 (6.0, 0.30)		
45–69	105 (31.8, -0.23)	57 (33.9, 0.32)		
≥70	208 (63.0, 0.23)	101 (60.1, -0.32)		
**Gender:**			0.924	0.004
Male	214 (64.9, -0.03)	110 (65.5, 0.05)		
Female	115 (34.8, 0.05)	58 (34.5, -0.06)		
Other	1 (0.3)*	0 (0.0)*		
**Education:**			**0.010**	0.151
Not completed primary school	11 (3.3, -0.08)	6 (3.6, 0.11)		
Completed primary school	73 (22.2, -1.61)	60 (35.7, 2.26)		
Completed secondary school	145 (43.9, 0.50)	65 (38.7, -0.69)		
Completed university	101 (30.6, 1.00)	37 (22.0, -1.40)		
**Hazardous alcohol use:**			0.052	0.087
No	250 (75.8, -0.53)	140 (83.3, 0.74)		
Yes	80 (24.2, 1.00)	28 (16.7, -1.40)		

*Gender=’other’ omitted from chi-square test, *n* = 497

The most widely accessed health information source was newspapers (32.9%), followed by television (29.2%), healthcare staff (13.4%), friends/family (11.8%), social media (8.9%) and websites (3.8%). In the majority of cases (77.9%), health information sources were reported to convey mixed messages about moderate drinking, i.e., that alcohol can be both good and bad for the heart (Table 2). According to participants, it was rare for media sources to report a consistent message that moderate drinking is bad for the heart (newspapers = 3.2%; television = 4.5%). Participants who reported hearing about cardiovascular effects of moderate alcohol consumption from healthcare staff indicated that most staff (66.7%) conveyed mixed messages about the effects of alcohol, while a minority (21.6%) had informed patients that alcohol is bad for the heart.

**Table 2:** Health information sources and reported messages about moderate alcohol consumption (*N* = 364)

Information source	Accessed by patient, n (%)	Message about moderate drinking and heart health, *n* (%)			Don’t know
		Good for heart	Both good and bad for heart	Bad for heart	
Healthcare staff	51 (13.4)	2 (3.9)	34 (66.7)	11 (21.6)	4 (7.8)
Newspaper	125 (32.9)	12 (9.6)	104 (83.2)	4 (3.2)	5 (4.0)
Television	111 (29.2)	10 (9.0)	91 (82.0)	5 (4.5)	5 (4.5)
Friends/family	45 (11.8)	3 (6.7)	36 (80.0)	4 (8.9)	2 (4.4)
Social media	34 (8.9)	0 (0.0)	31 (91.2)	3 (8.8)	0 (0.0)
Website	14 (3.8)	0 (0.0)	0 (0.0)	14 (100.0)	0 (0.0)
Total	380*	27 (7.1)	296 (77.9)	41 (10.8)	16 (4.2)

*Indicates total information sources accessed (participants were asked to indicate one or more sources)

‘Don’t know’ responses (*n* = 39) were excluded, ‘other’ (unspecified) information sources (*n* = 38) are not shown

Overall, exposure to information sources that reported that moderate drinking is good for the heart, or mixed messages about moderate alcohol consumption, was associated with increased odds of hazardous alcohol use (Table 3). In multiple logistic regression models, odds ratios for hazardous alcohol use were significantly elevated after adjustments for age group, gender and education (OR = 1.67, 95%CI = 1.02–2.74).

**Table 3:** Association between exposure to health information sources suggesting healthy heart effects and hazardous alcohol use; logistic regression models (*N* = 498)

	OR (95% CI)			
	**Univariate**	**Model 1**	**Model 2**	**Model 3**
**Exposure status:**				
Not exposed to healthy heart effect	Ref	Ref	Ref	Ref
Exposed to healthy heart effect	1.60 (0.99–2.58)	**1.67 (1.03–2.72)**	**1.69 (1.04–2.76)**	**1.67 (1.02–2.74)**
**Age group:**				
18–44	Ref	Ref	Ref	Ref
45–69	2.20 (0.79–6.13)	2.20 (0.79–6.15)	2.14 (0.76–6.00)	2.31 (0.81–6.58)
≥70	0.83 (0.30–2.29)	0.81 (0.29–2.25)	0.79 (0.28–2.21)	0.85 (0.30–2.38)
Gender*:				
Male	Ref	Ref	Ref	Ref
Female	0.62 (0.39–1.00)		0.63 (0.39–1.01)	**0.60 (0.37–0.98)**
Education:				
Not completed primary school	Ref	Ref	Ref	Ref
Completed primary school	**0.30 (0.10–0.87)**			**0.27 (0.09–0.82)**
Completed secondary school	0.41 (0.15–1.14)			**0.30 (0.10–0.89)**
Completed university	0.41 (0.15–1.18)			**0.33 (0.11–0.98)**

Model 1 adjusts for age group (Cox & Snell R²=0.045, Nagelkerke R²= 0.069)

Model 2 adjust for age group and gender (Cox & Snell R²=0.052, Nagelkerke R²=0.080)

Model 3 adjusts for age group, gender and education (Cox & Snell R²=0.062, Nagelkerke R²= 0.095)

*Gender=’other’ omitted from logistic regression analyses, *n* = 497

Bold text indicates *p*-value < 0.05

Sensitivity analyses, consisting of ordinal logistic regression models, produced results that were materially the same as those of the main analyses (Supplementary material 4).

## Discussion

This is, as far as we are aware, the first study on health information about cardiovascular health and alcohol use outside of a US context. Our findings show that about a third of patients in cardiology services – particularly those with a higher level of education – described being exposed to information sources that reported healthy heart effects with moderate alcohol consumption. Most participants reported accessing health information about alcohol from media sources, rather than from health professionals. There was a strong tendency for health information sources to convey mixed or conflicting messages about the cardiovascular effects of alcohol – irrespective of information source. Exposure to messages that moderate drinking has healthy heart effects, or mixed messages about moderate drinking, was associated with increased odds of hazardous alcohol use.

We found that many patients with cardiovascular disease report being exposed to suggestions of a healthy heart effect, an observation consistent with those of a US-based survey [10]. Our finding that older patients were more likely to have heard that moderate alcohol consumption can affect the heart is in line with results from the eHeart Study, a US-based survey of 5,582 people with CVDs [19]. This may suggest either that such reports tend to target older people, that older people are more perceptive to this type of message, or that information about alcohol and the heart has been less widely reported in recent years.

Overall, our finding that less than half of participants had heard that moderate alcohol consumption can affect the heart is consistent with limited public awareness of the wider risks of alcohol use. It has long been established, for example, that alcohol consumption increases the risk of cancer, yet public awareness of this link is low [25], particularly among men and those with lower education [26, 27]. In our study, there was no association between gender or educational level and awareness that alcohol can affect the heart. However, among participants who had heard of cardiovascular effects, those with lower education were less likely to have heard that alcohol is good for the heart, in similarity to the eHeart Study [19]. Evidence suggests that wine is more frequently perceived as heart healthy than spirits or beer [28]. Given that that regular wine consumption may be associated with higher socioeconomic position [29], it is possible that beverage preferences may have contributed to the differences we observed in exposure to reports of healthy heart effects according to educational level [19]. Knowledge of alcohol’s cardiovascular effects may also be linked to wider awareness of the risks of alcohol. In a recent study, those aware that alcohol use increases the risk of heart disease were more likely to also be aware of the link between alcohol and cancer [28], suggesting that risk perceptions surrounding alcohol use and CVDs may generalize to other negative health consequences.

We found that many patients with CVDs access health information about alcohol from media sources. About a quarter of reports described as suggesting a healthy heart effect were from television, while a third were in newspapers. These findings are consistent with those of Medling et al. [10]. In their study, 61% of participants had heard of healthy heart effects via television and 21% from newspapers. Further evidence regarding the importance of media sources in conveying public health information about alcohol can be found in the Alcohol Toolkit study – a survey of the general UK population [24]. The Alcohol Toolkit study assessed respondents’ awareness and knowledge of national alcohol guidelines. On average, across seven repeated surveys, television and radio were the most accessed sources of information about the alcohol guidelines, followed by newspapers and magazines.

The theory of lay epidemiology proposes that media and informal information sources are influential in shaping individuals’ health beliefs [17, 18]. Our findings suggest that reports of healthy heart effects are encountered in social contexts with friends and family, although somewhat less frequently than via television reports and newspaper articles. Lay epidemiology theory suggests that these conversations may alter how individuals understand and respond to the perceived risks and benefits associated with regular, low-level alcohol consumption. The HBM further proposes that any changes to individuals’ beliefs and expectations about the health effects of alcohol may subsequently lead to changes in behaviour [15]. While our study does not provide evidence of causality, lay epidemiology and the HBM offer possible theoretical explanations for our finding of increased odds of hazardous alcohol use among participants exposed to reports of healthy heart effects; namely that exposure to reports of protective cardiovascular effects may have influenced patients with CVDs to perceive moderate drinking as being safe, or even therapeutic, and thus increased the likelihood that exposed participants engage in regular alcohol consumption amounting to hazardous drinking.

Our finding that health information sources often convey mixed messages about alcohol may have additional implications for how patients with CVDs perceive risks associated with drinking. Exposure to reports about alcohol’s harmful effects may trigger negative emotions among drinkers, leading to biased appraisal – or ‘defensive processing’ – of conflicting health information [30]. Defensive processing may have led participants towards unrealistically optimistic interpretations of mixed messages about alcohol, causing them to favour reports of alcohol’s suggested positive effects. A related concept, known as cognitive dissonance, suggests that perceived inconsistency between information sources and participants’ own experiences of alcohol use may introduce an additional source of psychological stress [31]. To reduce cognitive dissonance, participants may have attributed more weight to reports of alcohol’s suggested positive effects. Overall, defensive processing and cognitive dissonance may have reduced the tendency for participants exposed to conflicting information to moderate their drinking, offering possible explanations for our finding of increased odds of hazardous alcohol use among those exposed to mixed messages about alcohol.

In similarity to the findings of Medling et al. [10], only one in eight participants in our study accessed information about alcohol directly from health professionals. Given the global consensus that any amount of alcohol consumption is unhealthy [32], it is troubling that health professionals were perceived to convey mixed messages about the cardiovascular effects of alcohol. Furthermore, our finding that information from clinicians was ambiguous regarding the health effects of alcohol suggests that opportunities for alcohol prevention – such as brief interventions [33] – are currently being missed. A recent Swedish study suggests that cardiology clinicians’ knowledge of key concepts in alcohol prevention, such as the definition of hazardous alcohol use, remains limited, which could contribute to staff communicating mixed messages to patients [34].

Our observation that exposure to suggested healthy heart effects, or mixed messages about alcohol, was associated with increased odds of hazardous drinking is in keeping with findings from the US [19]. However, studies on awareness of the link between alcohol and cancer in the general population have failed to demonstrate similar associations [18, 28]. While it is not possible to directly compare these findings due to differences in the definitions and measures used for hazardous alcohol use and study populations, existing studies suggest that the association we observed with hazardous alcohol use may be specific to CVD.

While controversy, or a lack of scientific consensus, may have contributed to mixed messages in earlier reports about alcohol’s cardiovascular effects, the influence of vested commercial interests on ambiguous reporting should also be considered [35]. Globally, the activity of the alcoholic beverage industry in promoting mixed messages about the health effects of alcohol is emphasized in the WHO’s 2023 guide to journalists for reporting on alcohol [32], with commercial activities increasingly recognized as key social determinants of health [36, 37]. Our findings relate specifically to the Swedish context, in which alcohol trade is subject to state regulation; Swedish legislation prohibits the marketing of any alcoholic beverages on public television and radio and a government monopoly is responsible for the retail sale of alcoholic beverages [38]. It is thus possible that the impact of private sector activities on how alcohol’s health effects are reported may be greater in countries with less restrictive alcohol policies [39–41].

Finally, our finding that participants perceived mixed messages in reports of alcohol’s health effects may have implications for policy implementation. While our findings are from Sweden, similar results have been reported elsewhere [10, 19, 28]. Evidence suggests that public support for evidence-based alcohol harm-reduction policies is associated with knowledge of health risks, particularly the link between alcohol and cancer [42]. Ambiguous reporting of the health effects of alcohol use may thus present a barrier to effective public health policy. Overall, evidence supports a comprehensive approach to reducing population-level alcohol harm, including interventions to increase public awareness around the risks of alcohol use, such as mass media campaigns [43, 44], health warning labels on alcoholic beverages [45], regulating alcohol marketing [46], and providing brief interventions during routine healthcare interactions [47].

### Strengths and limitations

Study strengths included recruitment of consecutive patients where possible and an acceptable response rate and sample size, permitting analysis of sociodemographic covariates such as educational level. While our sample may not be nationally representative, an additional strength was the collection of data from three heterogenous regions in Sweden. We identified the following limitations: This study was cross-sectional in design and is thus unable to determine the direction of the associations observed, or to establish causality. We did not ask participants when information sources had been accessed – it is therefore possible that many of the healthcare interactions and media sources reported were historical. Nor did we assess whether the participants stated that they believed in the messages around heart health. Regarding the study outcome, we did not assess reasons for abstaining among participants who reported not drinking alcohol during past year. While our sensitivity analysis generated results consistent with the study’s main findings, we note that participants may have avoided drinking alcohol for medical reasons, including CVDs. It is also possible that survey responses regarding the study exposure were subject to recall bias. Additionally, despite countermeasures such as explaining that survey completion was anonymous, we acknowledge that self-reported alcohol screening methods tend to underestimate hazardous alcohol use as a result of social desirability bias [48] and other factors.

## Conclusions

This study suggests that many patients in cardiology care in Sweden access health information about alcohol from media sources. Participants reported that health information sources often conveyed mixed messages about the cardiovascular effects of alcohol. Exposure to information suggesting that moderate drinking has protective cardiovascular – or “healthy heart” – effects, or mixed messages about the cardiovascular effects of alcohol, was associated with increased odds of hazardous alcohol use. Findings highlight a need for clear and consistent messages about the health effects of alcohol, both from media sources and health professionals.

### Electronic supplementary material

Below is the link to the electronic supplementary material.

Supplementary Material 1

## Data Availability

The datasets used and/or analysed during the current study are available from the corresponding author on reasonable request.
