# Supplementary material for: Mixed messages? Exposure to reports about alcohol’s suggested cardiovascular effects and hazardous alcohol use: a cross-sectional study of patients in cardiology care
Source: BMC Public Health. 2024 May 13;24:1302. doi: 10.1186/s12889-024-18783-5 (PMC11092105; doi:10.1186/s12889-024-18783-5)
Supplement: Supplementary file 1 — Supplementary Material 1 [file 12889_2024_18783_MOESM1_ESM.docx]

**Supplementary materials**

**Supplementary material 1:**

STROBE Statement—Checklist of items that should be included in reports of ***cross-sectional studies***

|  | Item No | Recommendation | Page number |
| --- | --- | --- | --- |
| **Title and abstract** | 1 | (*a*) Indicate the study’s design with a commonly used term in the title or the abstract | 1 |
|  |  | (*b*) Provide in the abstract an informative and balanced summary of what was done and what was found | 3-4 |
| Introduction | | |  |
| Background/rationale | 2 | Explain the scientific background and rationale for the investigation being reported | 5-7 |
| Objectives | 3 | State specific objectives, including any prespecified hypotheses | 7 |
| Methods | | |  |
| Study design | 4 | Present key elements of study design early in the paper | 7 |
| Setting | 5 | Describe the setting, locations, and relevant dates, including periods of recruitment, exposure, follow-up, and data collection | 8 |
| Participants | 6 | (*a*) Give the eligibility criteria, and the sources and methods of selection of participants | 8 |
| Variables | 7 | Clearly define all outcomes, exposures, predictors, potential confounders, and effect modifiers. Give diagnostic criteria, if applicable | 9 |
| Data sources/ measurement | 8* | For each variable of interest, give sources of data and details of methods of assessment (measurement). Describe comparability of assessment methods if there is more than one group | 10 |
| Bias | 9 | Describe any efforts to address potential sources of bias | 10 |
| Study size | 10 | Explain how the study size was arrived at | 10 |
| Quantitative variables | 11 | Explain how quantitative variables were handled in the analyses. If applicable, describe which groupings were chosen and why | 11 |
| Statistical methods | 12 | (*a*) Describe all statistical methods, including those used to control for confounding | 11 |
|  |  | (*b*) Describe any methods used to examine subgroups and interactions | N/A |
|  |  | (*c*) Explain how missing data were addressed | 11-12 |
|  |  | (*d*) If applicable, describe analytical methods taking account of sampling strategy | N/A |
|  |  | (*e*) Describe any sensitivity analyses | 12 |
| Results | | |  |
| Participants | 13* | (a) Report numbers of individuals at each stage of study—eg numbers potentially eligible, examined for eligibility, confirmed eligible, included in the study, completing follow-up, and analysed | 12, Fig. 1 |
|  |  | (b) Give reasons for non-participation at each stage | Fig. 1 |
|  |  | (c) Consider use of a flow diagram | Fig. 1 |
| Descriptive data | 14* | (a) Give characteristics of study participants (eg demographic, clinical, social) and information on exposures and potential confounders | 12-13, Table 1 |
|  |  | (b) Indicate number of participants with missing data for each variable of interest | Fig. 1 |
| Outcome data | 15* | Report numbers of outcome events or summary measures | 12-13 |
| Main results | 16 | (*a*) Give unadjusted estimates and, if applicable, confounder-adjusted estimates and their precision (eg, 95% confidence interval). Make clear which confounders were adjusted for and why they were included | 14, Table 3 |
|  |  | (*b*) Report category boundaries when continuous variables were categorized | Table 1, Table 3 |
|  |  | (*c*) If relevant, consider translating estimates of relative risk into absolute risk for a meaningful time period | N/A |
| Other analyses | 17 | Report other analyses done—eg analyses of subgroups and interactions, and sensitivity analyses | 14 |
| Discussion | | |  |
| Key results | 18 | Summarise key results with reference to study objectives | 14-15 |
| Limitations | 19 | Discuss limitations of the study, taking into account sources of potential bias or imprecision. Discuss both direction and magnitude of any potential bias | 20-21 |
| Interpretation | 20 | Give a cautious overall interpretation of results considering objectives, limitations, multiplicity of analyses, results from similar studies, and other relevant evidence | 15-21 |
| Generalisability | 21 | Discuss the generalisability (external validity) of the study results | 19-20 |
| Other information | | |  |
| Funding | 22 | Give the source of funding and the role of the funders for the present study and, if applicable, for the original study on which the present article is based | 21-22 |

*Give information separately for exposed and unexposed groups.

**Note:** An Explanation and Elaboration article discusses each checklist item and gives methodological background and published examples of transparent reporting. The STROBE checklist is best used in conjunction with this article (freely available on the Web sites of PLoS Medicine at http://www.plosmedicine.org/, Annals of Internal Medicine at http://www.annals.org/, and Epidemiology at http://www.epidem.com/). Information on the STROBE Initiative is available at [www.strobe-statement.org](http://www.strobe-statement.org).

**Supplementary material 2**

**Questionnaire**

Background questions

How do you define your gender identity?

Male

Female

Other

How old are you? ______ years

What is the highest level of education that you have completed?

Have not completed primary school or equivalent

Completed primary school

Completed secondary school (gymnasium) or equivalent

Completed university or other higher education

Does anyone in your family suffer from heart or blood vessel (cardiovascular) disease?

Yes

No

Don’t know

Which diagnosis is the reason for your contact with the hospital today?

Heart attack (also known as a myocardial infarction)

Angina

Abnormal heart rhythm

Heart failure

Stroke

Other

Don’t know

If other 🡪 please write the diagnosis below

Alcohol

Have you drunk alcohol during the last 12 months?

Yes

No

If yes 🡪 AUDIT-C

If no 🡪 Have you drunk alcohol earlier in life? (Yes/no)

AUDIT-C questionnaire

| Below are some questions about your alcohol habits during the past year. Please answer them carefully and honestly by marking the option by applies for you. | |
| --- | --- |
| How often do you have a drink containing alcohol? | |
|  | Never |
|  | Monthly or less |
|  | 2-4 times per month |
|  | 2-3 times per week  Daily or almost everyday |
| How many drinks containing alcohol (see example) do you have on a typical day when you are drinking? | |
|  | 1-2 |
|  | 3-4 |
|  | 5-6 |
|  | 7-9 |
|  | 10 or more |
| How often do you have six or more drinks on the same occasion? | |
|  | Never |
|  | Less than monthly |
|  | Monthly |
|  | Weekly |
|  | Daily or almost daily |

Your experiences with alcohol and health

Have you ever heard that "moderate alcohol consumption" can affect your heart?

No

Don’t know

Yes

If yes 🡪 in what way?

Bad for the heart

Both that it can be bad and good for the heart

Good for the heart

Don’t know

Where did you hear this? (choose all that apply):

Doctor / other healthcare staff (yes/no, if yes: good or bad for the heart)

Newspaper (yes/no, if yes: good or bad for the heart)

TV (yes/no, if yes: good or bad for the heart)

Friend / family (yes/no, if yes: good or bad for the heart)

Social media (Facebook, Instagram etc) (yes/no, if yes: good or bad for the heart)

Website (yes/no, if yes: good or bad for the heart)

Other

Don’t know

**Supplementary material 3:**

Characteristics of study participants, by exposure to information sources reporting that moderate alcohol consumption can affect the heart, (N=1025)

|  | Heard that moderate drinking can affect the heart, n=498 (48.6%): n (%, standardised residual) | Not heard that moderate drinking can affect the heart* n=527 (51.4%): n (%, standardised residual) | *p-value* | Cramér's V |
| --- | --- | --- | --- | --- |
| Age group: |  |  | **.042** | .079 |
| 18–44 | 27 (5.4, -1.13) | 42 (8.0, 1.10) |  |  |
| 45–69 | 162 (32.5, -0.91) | 196 (37.2, 0.88) |  |  |
| ≥70 | 309 (62.1, 1.08) | 289 (54.8, -1.05) |  |  |
| Gender: |  |  | .873 | .016 |
| Male | 324 (65.1, -0.22) | 351 (66.6, 0.21) |  |  |
| Female | 173 (34.7, 0.30) | 175 (33.2, -0.29) |  |  |
| Other** | 1 (0.2) | 1 (0.2) |  |  |
| Education: |  |  | .577 | .044 |
| Not completed primary school | 17 (3.4, -0.45) | 22 (4.2, 0.44) |  |  |
| Completed primary school | 133 (26.7, -0.22) | 146 (27.7, 0.21) |  |  |
| Completed secondary school | 210 (42.2, -0.32) | 232 (44.0, 0.32) |  |  |
| Completed university | 138 (27.1, 0.82) | 127 (24.1, -0.79) |  |  |
| Hazardous alcohol use: |  |  | **.005** | .088 |
| No | 390 (78.3, 1.03) | 372 (70.6, -0.10) |  |  |
| Yes | 108 (21.7, -1.75) | 155 (29.4, 1.70) |  |  |
|  |  |  |  |  |

*Or unsure whether heard that moderate drinking can affect the heart

**Gender=’other’ omitted from chi-square test, n=1023

1. **Supplementary material 4. Sensitivity analyses: Association between exposure to health information sources suggesting healthy heart effects and hazardous alcohol use using three-level alcohol use variable; ordinal logistic regression models (N=498)**

|  | OR (95% CI) | | | |
| --- | --- | --- | --- | --- |
|  | **Univariate** | **Model 1** | **Model 2** | **Model 3** |
| Exposure status: |  |  |  |  |
| Not exposed to healthy heart effect | Ref | Ref | Ref | Ref |
| Exposed to healthy heart effect | **1.69 (1.19–2.41**) | **1.74 (1.22–2.48)** | **1.75 (1.22–2.49)** | **1.68 (1.18–2.41)** |
| Age group: |  |  |  |  |
| 18–44 | Ref | Ref | Ref | Ref |
| 45–69 | 1.68 (0.79–3.58) | 1.67 (0.78–3.58) | 1.65 (0.77–3.54) | 1.82 (0.84–3.94) |
| ≥70 | 0.80 (0.38–1.65) | 0.78 (0.37–1.62) | 0.77 (0.37–1.62) | 0.86 (0.41–1.80) |
| Gender*: |  |  |  |  |
| Male | Ref | Ref | Ref | Ref |
| Female | **0.57 (0.40–0.81)** |  | **0.57 (0.40–0.82)** | **0.60 (0.37–0.98)** |
| Education: |  |  |  |  |
| Not completed primary school | Ref | Ref | Ref | Ref |
| Completed primary school | **0.28 (0.11–0.73)** |  |  | **0.25 (0.09–0.69)** |
| Completed secondary school | 0.43 (0.17–1.11) |  |  | **0.32 (0.12–0.85)** |
| Completed university | 0.49 (0.19–1.27) |  |  | 0.38 (0.13–1.02) |

Model 1 adjusts for age group (McFadden's R²=0.024)

Model 2 adjust for age group and gender (McFadden's R²=0.033)

Model 3 adjusts for age group, gender and education (McFadden's R²=0.042)

*Gender=’other’ omitted from analyses, n=497

Bold text indicates *p*-value<.05
